# Recent Advances in Multimodal Diagnosis and Precision Management of Myocarditis

**DOI:** 10.31083/RCM51061

**Published:** 2026-10-14

**Authors:** Weiting Cai, Jing Zhao, Andong Lu, Zheng Zhang

**Affiliations:** ^1^Department of Coronary Care Unit, The First Hospital of Lanzhou University, 730013 Lanzhou, Gansu, China; ^2^Gansu Province Clinical Research Center for Cardiovascular Diseases, 730000 Lanzhou, Gansu, China; ^3^Heart Center, The First Hospital of Lanzhou University, 730013 Lanzhou, Gansu, China

**Keywords:** myocarditis, magnetic resonance imaging, biomarkers, endomyocardial biopsy, immune checkpoint inhibitors, immunomodulation, extracorporeal membrane oxygenation, risk stratification

## Abstract

Myocarditis is an inflammatory disease of the myocardium caused by infectious, autoimmune, or toxic triggers; meanwhile, myocarditis remains a persistent clinical challenge due to the heterogeneous nature and often nonspecific presentation of myocarditis. Recent advances in cardiac magnetic resonance (CMR), particularly quantitative tissue characterization techniques, together with improvements in molecular diagnostic methods, have substantially strengthened the accuracy of noninvasive diagnosis. Concurrently, management strategies are increasingly shifting toward etiology-directed approaches, including targeted immunomodulatory therapies combined with optimized supportive care, thereby contributing to improved clinical outcomes. This review summarizes key developments in myocarditis over the past five years, with a focus on progress in multimodal diagnostic evaluation and precision management. We first outline current perspectives on etiology and clinical phenotypes, then examine the complementary roles of quantitative CMR imaging and endomyocardial biopsy in diagnostic decision-making. Recent advances in the management of major clinical subtypes, including viral myocarditis, immune checkpoint inhibitor-associated myocarditis, giant cell myocarditis, and pediatric myocarditis, are then discussed. Indeed, by integrating emerging clinical evidence, we highlight the evolving diagnostic and therapeutic framework, identify remaining challenges, and propose future directions to improve risk stratification and individualized patient care.

## 1. Introduction

Myocarditis encompasses a heterogeneous group of inflammatory myocardial disorders caused by infectious agents, immune-mediated injury, or drug-related toxicity. Clinical manifestations vary widely, ranging from asymptomatic disease to acute heart failure and sudden cardiac death, which continues to complicate timely recognition and standardized management [1,2]. Viral infection remains the predominant etiology, particularly enteroviruses, parvovirus B19, adenovirus, and SARS-CoV-2 (Severe Acute Respiratory Syndrome Coronavirus 2), although the relative contribution varies across populations [3,4,5]. Myocardial injury reflects a combination of direct viral cytotoxicity and maladaptive host immune responses [3,4]. In parallel, the expanding use of immune checkpoint inhibitors in oncology has led to increasing recognition of treatment-related immune-mediated myocarditis, introducing new challenges in diagnosis and management [6].

Diagnostic evaluation has traditionally relied on clinical presentation, electrocardiographic findings, circulating biomarkers, and endomyocardial biopsy [7]. Although biopsy provides histopathological confirmation, its invasive nature and sampling variability limit widespread clinical use [7]. Advances in cardiovascular imaging, particularly cardiac magnetic resonance (CMR), have substantially reshaped the diagnostic landscape [8,9,10,11]. Quantitative tissue characterization techniques—including T1 and T2 mapping and late gadolinium enhancement—enable sensitive detection of myocardial edema, inflammation, and fibrosis, thereby improving disease classification and prognostic assessment [9,10,11]. The integration of artificial intelligence–assisted image analysis may improve diagnostic performance and has shown potential in preliminary studies, although further prospective validation is required before routine clinical implementation [12,13].

Therapeutic strategies are evolving from largely supportive care toward etiology-directed and immunomodulatory approaches. Immunosuppressive therapy has demonstrated clinical benefit in selected patients with virus-negative immune-mediated myocarditis [14], whereas immune checkpoint inhibitor–associated myocarditis generally requires prompt initiation of high-dose corticosteroids, with additional immunomodulatory therapies considered in refractory cases [15,16]. Nevertheless, current therapeutic recommendations are still largely based on observational evidence, highlighting the need for adequately powered randomized clinical trials [14,15].

Overall, contemporary management of myocarditis increasingly depends on the integration of histopathological findings with advanced multimodal imaging, particularly CMR-based evaluation [7,8]. Further multicenter studies are needed to refine diagnostic algorithms, optimize therapeutic strategies, and support individualized risk stratification aimed at improving long-term clinical outcomes.

## 2. Etiology, Pathophysiology, and Clinical Presentation

### 2.1 Etiologic Classification of Myocarditis

#### 2.1.1 Viral Myocarditis

Viral infection remains the predominant cause of myocarditis, with enteroviruses, adenovirus, and SARS-CoV-2 among the most frequently implicated pathogens. Myocardial injury may result from direct viral cytotoxicity as well as from dysregulated innate and adaptive immune responses, leading to persistent inflammation, myocardial fibrosis, and ventricular remodeling. In susceptible individuals, these processes may ultimately progress to dilated cardiomyopathy or chronic heart failure [17,18]. The COVID-19 pandemic has renewed attention to virus-associated myocardial injury, as SARS-CoV-2 enters cardiomyocytes through the angiotensin-converting enzyme 2 (ACE2) receptor and can trigger systemic inflammatory activation [4,19]. Despite increasing mechanistic insight, treatment of viral myocarditis remains largely supportive, while disease-specific antiviral and immunomodulatory approaches are still under investigation [20].

#### 2.1.2 Autoimmune and Immune-Mediated Myocarditis

Immune dysregulation represents another major pathogenic pathway. Immune checkpoint inhibitor (ICI)–associated myocarditis and myocarditis related to systemic autoimmune diseases are increasingly recognized in clinical practice. By enhancing T-cell activation, ICIs improve antitumor immunity but may also precipitate immune-mediated myocardial injury, a rare complication associated with substantial mortality [21,22]. High-dose corticosteroids remain first-line therapy, whereas adjunctive treatments such as plasma exchange and other immunomodulatory strategies may be considered in refractory cases [23,24]. Myocardial involvement is also observed in systemic autoimmune disorders such as systemic lupus erythematosus, rheumatoid arthritis, and inflammatory myopathies, where autoantibodies, activated T cells, and cytokine signaling networks contribute to myocardial inflammation. Genetic variants affecting immune regulation, including mutations in* IFIH1 *encoding the viral RNA sensor MDA5 (melanoma differentiation-associated protein 5), may further increase susceptibility to autoimmune myocarditis. Clinical management therefore requires integrated assessment of cardiac involvement together with treatment of the underlying systemic disease [25,26].

#### 2.1.3 Drug-Induced and Rare Forms

Certain less common subtypes, including giant cell myocarditis and eosinophilic myocarditis, are characterized by rapid progression and unfavorable outcomes. Giant cell myocarditis frequently presents with fulminant heart failure or malignant arrhythmias and usually requires histopathological confirmation by endomyocardial biopsy. Early initiation of immunosuppressive therapy is recommended, although a proportion of patients ultimately require heart transplantation. Eosinophilic myocarditis is often associated with parasitic infection, drug hypersensitivity, or immune disorders and typically requires prompt etiologic treatment combined with immunosuppression [27]. Drug-related myocarditis, although relatively uncommon, is clinically important and may occur in association with immune checkpoint inhibitors, anti-inflammatory agents, or toxic exposures; early recognition and discontinuation of the offending agent remain essential for improving outcomes [28,29].

### 2.2 Clinical Presentation and Pathophysiology

The clinical spectrum of myocarditis is highly heterogeneous, ranging from asymptomatic myocardial injury to acute heart failure, malignant arrhythmias, and cardiogenic shock. Sudden cardiac death occurs more frequently in younger populations, highlighting the importance of early recognition. Circulating biomarkers, including cardiac troponins and natriuretic peptides, together with CMR, provide essential information for estimating myocardial injury burden and disease severity [30,31]. Clinical manifestations and outcomes vary substantially according to disease etiology, host immune response, and patient characteristics [31,32,33].

From a pathophysiological perspective, myocarditis reflects a dynamic interplay between initial myocardial injury and subsequent immune-mediated inflammation. Viral infection or toxic exposure triggers cardiomyocyte damage, followed by activation of innate and adaptive immune responses that may amplify myocardial injury. In some patients, persistent immune activation contributes to myocardial fibrosis and adverse ventricular remodeling, which are key determinants of long-term prognosis [24,26]. These mechanisms provide a conceptual framework for risk stratification and support the development of targeted therapeutic strategies.

## 3. Advances in Diagnostic Strategies

### 3.1 Laboratory Biomarkers

Laboratory biomarkers remain fundamental to the early detection and risk stratification of myocarditis, providing complementary information on myocardial injury, ventricular stress, and inflammatory activation. Cardiac troponins are widely used indicators of myocardial injury, although their prognostic value varies across etiologies. In ICI-associated myocarditis, persistent troponin elevation has been associated with a higher risk of MACE (major adverse cardiovascular events) [34]. High-sensitivity troponin assays may also assist in excluding myocarditis in selected low-risk settings, thereby reducing unnecessary downstream imaging [35].

Natriuretic peptides, including BNP (brain natriuretic peptide) and NT-proBNP (N-terminal pro B-type natriuretic peptide), primarily reflect ventricular loading conditions and are frequently elevated in severe or pediatric cases, where they correlate with intensive care admission and mortality risk [30]. Inflammatory markers such as CRP (C-reactive protein), ferritin, and D-dimer may reflect systemic inflammatory activity, but their specificity for myocarditis remains limited. Several exploratory biomarkers, including sST2 (soluble suppression of tumorigenicity 2), Siglec-5 (sialic acid-binding Ig-like lectin 5), and CD163 (cluster of differentiation 163), have been proposed for identifying high-risk or fulminant presentations, but external validation remains limited [36,37].

Viral nucleic acid testing, particularly from myocardial tissue when endomyocardial biopsy (EMB) is performed, may support etiologic classification in selected cases, including suspected parvovirus B19 or enterovirus-associated myocarditis [38,39]. In suspected immune-mediated myocarditis, autoantibody testing may provide supportive diagnostic information, primarily assisting in differential diagnosis rather than serving as a standalone diagnostic tool [40]. Novel molecular biomarkers, including circulating microRNAs, free light chains, and TNF-α (tumor necrosis factor-alpha), are currently being explored for their potential value in improving mechanistic understanding and future risk-stratification strategies [41,42,43,44]. Optimal clinical use of laboratory testing therefore relies on integration with imaging findings and longitudinal patient assessment rather than interpretation of individual markers in isolation.

### 3.2 Electrocardiography and Echocardiography

Electrocardiography (ECG) provides rapid detection of myocardial injury–related electrical abnormalities, although its findings are generally nonspecific. ST–T changes represent the most frequent pattern, and both ST-segment elevation and T-wave inversion are commonly observed in acute myocarditis across pediatric and adult populations, often correlating with troponin levels and disease severity [45,46]. In fulminant presentations, ventricular tachyarrhythmias, high-grade atrioventricular block, QRS (QRS complex) prolongation, and low-voltage complexes identify patients at increased risk of electrical instability. In ICI–associated myocarditis, sinus tachycardia, QTc (corrected QT interval) prolongation, and QRS abnormalities have similarly been linked to severe disease and adverse outcomes [47,48]. Fragmented QRS complexes may also correspond to regions of late gadolinium enhancement (LGE) detected by cardiac magnetic resonance (CMR), providing an additional diagnostic clue when interpreted within the appropriate clinical context [49].

Transthoracic echocardiography remains the first-line bedside imaging modality for evaluating cardiac structure, ventricular function, and hemodynamic status, particularly in patients presenting with acute chest pain or circulatory instability. Because left ventricular ejection fraction (LVEF) may remain preserved in early disease, strain-based techniques such as speckle-tracking echocardiography (STE) have become increasingly valuable for identifying subclinical systolic dysfunction. Reductions in global longitudinal or circumferential strain frequently parallel the distribution of myocardial injury detected by CMR-LGE and have been associated with adverse clinical outcomes, supporting their role in risk stratification and longitudinal follow-up [50,51,52,53]. Assessment of right ventricular function provides additional prognostic information in selected myocarditis phenotypes [54,55]. Together, ECG and echocardiography constitute the essential first step in the multimodal diagnostic pathway, guiding the selection of advanced imaging or endomyocardial biopsy when indicated.

### 3.3 Cardiac Magnetic Resonance Imaging (CMR)

CMR has become a central noninvasive modality for the evaluation of suspected myocarditis, enabling comprehensive assessment of myocardial inflammation and structural injury. The updated 2018 Lake Louise Criteria incorporate edema-sensitive sequences, including T2-weighted imaging and T2 mapping, together with tissue characterization techniques such as LGE, native T1 mapping, and extracellular volume (ECV) quantification. This multiparametric approach allows integrated evaluation of inflammatory activity, myocardial injury, and fibrosis, providing high diagnostic performance while reducing the need for endomyocardial biopsy in a substantial proportion of patients [56]. In the subacute or chronic phase, quantitative mapping techniques—particularly T2 mapping—can identify residual inflammatory activity, and interpretation is strengthened when integrated with circulating biomarkers such as troponin and CRP [11].

CMR phenotypes vary across etiologic subtypes. In ICI–associated myocarditis, structural abnormalities may be relatively subtle, yet T1 mapping and LGE frequently detect myocardial involvement and support risk stratification [57,58]. In contrast, COVID-19–related and vaccine-associated myocarditis more commonly demonstrate detectable myocardial edema and characteristic LGE patterns that assist in evaluating disease severity and recovery trajectories [59,60].

Recent advances in CMR feature-tracking strain analysis have further expanded functional assessment. Even in patients with preserved LVEF, reductions in global longitudinal strain (GLS) may reveal early myocardial dysfunction and provide incremental prognostic information beyond conventional CMR features [61]. Pediatric data also suggest that ventricular strain abnormalities may be detectable in children with myocarditis, although their prognostic role requires further validation [62]. Collectively, CMR provides an integrated platform for diagnosing myocarditis, characterizing disease phenotype, and estimating prognostic risk [56,61].

### 3.4 Endomyocardial Biopsy (EMB)

EMB remains the reference standard for histological and molecular confirmation of myocarditis, particularly when differentiation between infectious and immune-mediated myocardial injury is required or when initiation of immunosuppressive therapy is being considered. Histopathological analysis combined with immunohistochemistry and viral polymerase chain reaction (PCR) testing enables direct assessment of inflammatory infiltration, myocyte necrosis, and fibrosis, thereby supporting etiologic classification [63]. Although advances in CMR have reduced reliance on invasive testing in many patients, EMB continues to play a pivotal role in fulminant myocarditis, rapidly progressive disease, treatment-refractory cases, and suspected giant cell or eosinophilic myocarditis, where tissue findings frequently guide therapeutic decision-making and prognostic evaluation [64,65].

The main limitations of EMB relate to procedural risk and sampling variability. Potential complications include bleeding, myocardial perforation, and arrhythmias, with event rates influenced by operator experience, procedure duration, and patient condition. Observational studies suggest that elevated BNP levels and prolonged procedural time may be associated with a higher risk of short-term complications, whereas early biopsy in appropriately selected patients may improve diagnostic efficiency and clinical outcomes [66,67]. Variability in diagnostic frameworks—including the continued use of both Dallas histological criteria and ESC (European Society of Cardiology) immunohistochemical standards—further underscores the need for improved standardization [68,69]. Accordingly, EMB is best reserved for patients in whom clinical management depends on tissue confirmation rather than being applied routinely to all suspected cases (Fig. 1).

**Fig. 1.**
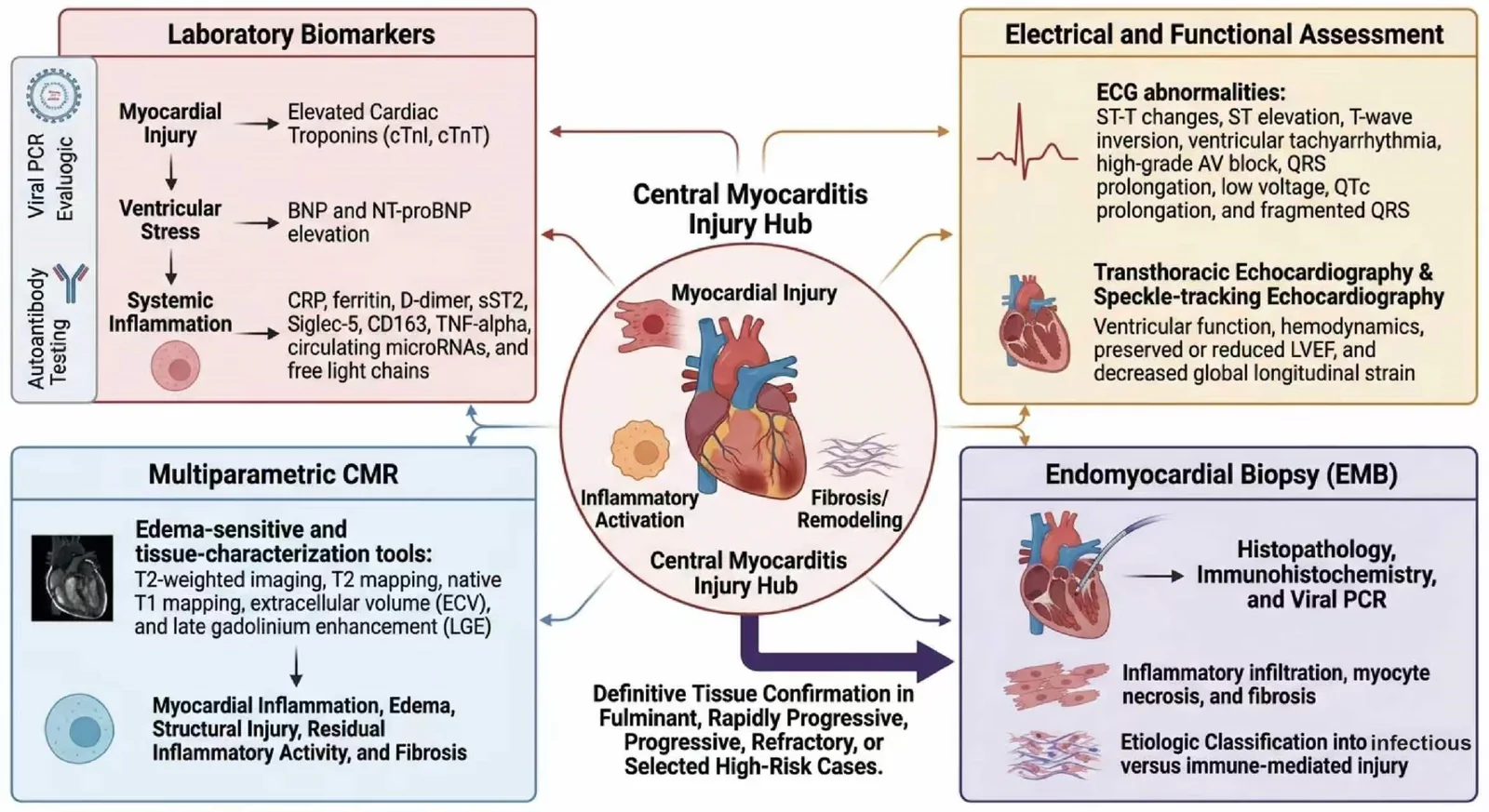
**Multimodal diagnostic framework for myocarditis**. Myocarditis is characterized by myocardial injury, inflammatory activation, and fibrosis/remodeling, which are reflected by circulating biomarkers and assessed through complementary diagnostic modalities. Laboratory biomarkers indicate myocardial injury, ventricular stress, and systemic inflammation, while electrocardiography (ECG) and echocardiography provide electrical and functional evaluation. Cardiac magnetic resonance (CMR) enables noninvasive characterization of myocardial inflammation, edema, structural injury, and fibrosis. Endomyocardial biopsy (EMB) remains the definitive method for tissue confirmation and etiologic classification in selected high-risk cases. cTnI, cardiac troponin; IcTnT, cardiac troponin T; BNP, brain natriuretic peptide; NT-proBNP, N-terminal pro B-type natriuretic peptide; CRP, C-reactive proteins; ST2, soluble suppression of tumorigenicity 2; Siglec-5, sialic acid-binding immunoglobulin-like lectin 5; CD163, cluster of differentiation 163; TNF-α, tumor necrosis factor-alpha.

## 4. Contemporary Therapeutic Strategies

### 4.1 Supportive and Mechanical Circulatory Support

Supportive therapy remains the foundation of myocarditis management, with priority given to stabilization of heart failure, arrhythmias, and hemodynamic compromise. Conventional heart failure therapies, including diuretics, vasodilators, and inotropic agents when required, are used to improve tissue perfusion and relieve congestion. Arrhythmias may range from conduction disturbances to malignant ventricular tachyarrhythmias; therefore, management should be individualized with pharmacologic therapy, cardioversion, or temporary or permanent pacing when appropriate. Temporary restriction of strenuous physical activity is generally recommended during the acute and recovery phases [70].

In patients with fulminant myocarditis or cardiogenic shock, pharmacological therapy alone may be insufficient to maintain adequate circulation. Mechanical circulatory support (MCS), particularly extracorporeal membrane oxygenation (ECMO), can provide bridging support while myocardial recovery occurs. Observational studies and registry data suggest that ECMO or other MCS strategies may improve short-term stabilization and survival in selected patients with fulminant myocarditis, although high-quality randomized evidence is lacking [70,71,72]. In selected cases, ventricular unloading strategies may further support myocardial recovery, but device selection should be individualized according to hemodynamic status and institutional expertise [71,72].

Supportive therapy should be integrated with etiologic management. Patients with immune-mediated myocarditis, especially ICI-associated cases, may require early immunosuppressive therapy in addition to life-support measures, whereas most viral myocarditis continues to rely primarily on supportive cardiac management because disease-specific antiviral treatments remain limited [17,70,72]. Collectively, standardized heart failure care, arrhythmia control, timely MCS deployment, and etiology-directed therapy constitute the central therapeutic framework for severe myocarditis [73,74].

### 4.2 Antiviral and Anti-Infective Therapy

Viral myocarditis is most associated with enteroviruses, particularly coxsackievirus B3, yet antiviral therapies or vaccines with proven clinical efficacy remain unavailable [75]. Several candidate compounds have demonstrated antiviral and anti-inflammatory effects in experimental studies. Agents such as baicalein, lithium salts, and other natural products inhibit viral replication through modulation of viral protease activity, apoptosis pathways, or activation of antiviral signaling cascades including the STING/TBK-1/IRF3 (stimulator of interferon genes/TANK-binding kinase 1/interferon regulatory factor 3) pathway, although current evidence remains largely preclinical [75,76,77]. Additional inhibitors targeting viral replication enzymes, including viral polymerase and protease inhibitors such as 4′-fluorouridine, have shown potential broad-spectrum activity against enteroviruses but require further clinical validation [78,79].

Therapeutic development is complicated by the diversity of viral pathogens and the persistence of immune-mediated myocardial inflammation after viral clearance, which limits the effectiveness of antiviral monotherapy. Consequently, combination approaches integrating antiviral and immunomodulatory therapies are increasingly being explored to simultaneously suppress viral replication and attenuate immune-mediated myocardial injury [79,80].

In SARS-CoV-2-associated myocarditis, disease-specific antiviral therapy has not been established, and clinical management remains centered on supportive care, with adjunctive antiviral or immunomodulatory treatments considered on a case-by-case basis [81]. Available evidence does not yet support consistent mortality benefits for specific antiviral or anti-inflammatory regimens in patients with COVID-19-related myocardial injury [82]. Future progress will depend on pathogen-specific antiviral development, better etiologic stratification, and carefully designed clinical studies evaluating combined antiviral and immune-modulating strategies.

### 4.3 Immunomodulatory Therapy

Immunomodulatory therapy represents a central component in the management of immune-mediated myocarditis, particularly in giant cell myocarditis and ICI–associated myocarditis. Current treatment strategies typically rely on high-dose corticosteroids, often combined with additional immunosuppressive agents such as cyclosporine to suppress T-cell activation and inflammatory cytokine signaling. In giant cell myocarditis, early combination immunosuppression is considered standard therapy and has been associated with improved cardiac function and survival [14]. Similarly, patients with virus-negative lymphocytic myocarditis may benefit from immunosuppressive therapy once active viral infection has been excluded [83,84]. In fulminant disease, combined immunosuppressive therapy and mechanical circulatory support may further improve short-term outcomes [85].

ICI-associated myocarditis, although relatively rare, carries substantial mortality risk. Management requires immediate discontinuation of the causative ICI agent and prompt initiation of high-dose corticosteroids [86]. Patients with inadequate response may require adjunctive therapies, including intravenous immunoglobulin, cyclosporine, methotrexate, or abatacept [86,87]. Emerging evidence implicates the (C-X-C motif chemokine receptor 3) CXCR3–CXCL9/10 (C-X-C motif chemokine ligand 9/10) signaling pathway in disease pathogenesis, providing potential targets for future immunomodulatory interventions [88]. In carefully selected low-grade cases, reintroduction or continuation of ICI therapy under close monitoring has been reported, but this approach requires individualized multidisciplinary risk assessment [89]. Severe arrhythmias or conduction disturbances may necessitate pacing support, and some abnormalities may resolve following effective immunosuppression [86]. In critically ill patients, ECMO remains an important adjunct to stabilize hemodynamics and facilitate myocardial recovery [70]. Despite these advances, optimal immunosuppressive regimens, treatment duration, and integration with mechanical circulatory support strategies remain to be defined in prospective studies [90].

### 4.4 Emerging Therapeutic Approaches

Therapeutic research in myocarditis is increasingly shifting beyond conventional immunosuppression toward targeted immune modulation and regenerative strategies. In ICI–associated myocarditis, adjunctive therapies such as plasma exchange, intravenous immunoglobulin, and cytokine-targeted biologic agents—including TNF-α and IL-6 (interleukin-6) inhibitors—have been explored in refractory cases [91]. However, current evidence is largely limited to case-based and observational reports, and their clinical efficacy remains to be established. Mechanistic studies have identified mitochondrial dysfunction and signaling pathways involving mTOR (mammalian target of rapamycin), GSK3β (glycogen synthase kinase-3 beta), PTPN11 (protein tyrosine phosphatase non-receptor type 11), and MFN2 (mitofusin 2) as potential contributors to disease pathogenesis, providing a rationale for future targeted interventions [92].

Regenerative approaches are also under active investigation. Experimental studies suggest that mesenchymal stem cells and cardiac progenitor cells may exert beneficial effects through immunomodulation, attenuation of fibrosis, and promotion of myocardial repair, although these findings remain largely preclinical [73]. Similarly, advances in engineered cellular therapies may improve tissue targeting and therapeutic precision, but clinical validation is still lacking. Molecularly targeted and nucleic acid-based strategies, including modulation of the TNF/TNFR1 (tumor necrosis factor receptor 1) signaling axis and microRNA-based interventions, have demonstrated anti-inflammatory and cytoprotective effects in experimental models, yet their translation into clinical practice remains uncertain [93,94,95]. Overall, while targeted immunotherapies, biologic agents, regenerative approaches, and molecular or gene-based interventions represent promising future directions, robust clinical evidence regarding their safety, efficacy, and long-term outcomes is still lacking (Fig. 2).

**Fig. 2.**
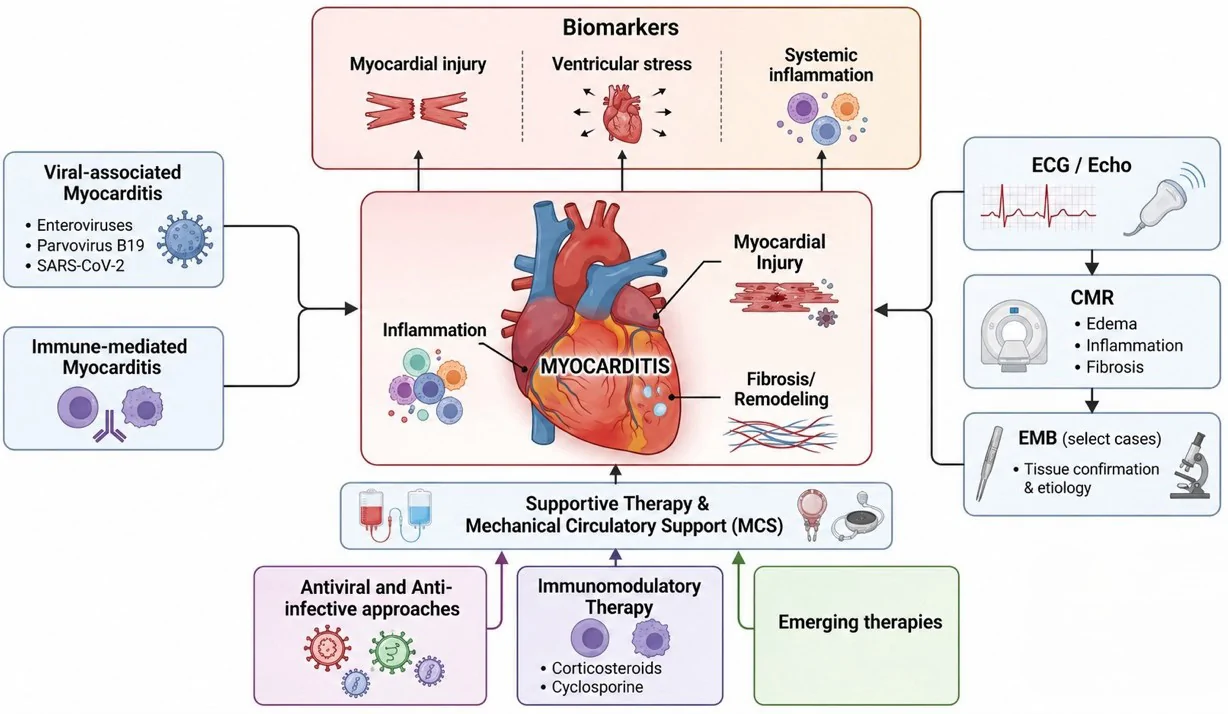
**Integrated overview of the major etiologies, diagnostic modalities, and therapeutic strategies in myocarditis**. Myocarditis may arise from viral-associated or immune-mediated mechanisms, leading to inflammatory infiltration, myocyte necrosis, myocardial edema, apoptosis, and fibrotic remodeling. These pathological changes are reflected by circulating biomarkers and evaluated through multimodal approaches, including ECG, echocardiography, CMR, and EMB. Current management is centered on supportive therapy and mechanical circulatory support (MCS), with additional antiviral, immunomodulatory, and emerging targeted therapies aimed at limiting myocardial injury and improving outcomes.

## 5. Myocarditis in Special Populations

### 5.1 Pediatric Myocarditis: Etiology and Clinical Features

Viral infection remains the predominant cause of pediatric myocarditis, with enteroviruses, parvovirus B19, and respiratory viruses representing the most frequently identified pathogens. Autopsy-based studies suggest that acute myocarditis accounts for approximately 3% of sudden deaths in children, with enteroviral infections being particularly prominent in neonatal cases. Early clinical recognition remains challenging because initial manifestations are often nonspecific or overlap with systemic infectious conditions [96]. Retrospective analyses further demonstrate that a broad range of respiratory pathogens—including rhinovirus, influenza virus, respiratory syncytial virus, adenovirus, and coronaviruses—may contribute to pediatric myocarditis, particularly among children younger than two years [97].

The COVID-19 pandemic has further reshaped the etiologic landscape. Multisystem inflammatory syndrome in children (MIS-C), typically developing several weeks after SARS-CoV-2 infection, has emerged as an important inflammatory cause of myocardial dysfunction and may progress to cardiogenic shock in severe cases [98,99,100]. Myocarditis following COVID-19 vaccination has also been reported, although incidence remains low and clinical outcomes are generally favorable [101]. In addition, immune-mediated conditions such as Kawasaki disease may involve the myocardium and contribute to coronary artery abnormalities or ventricular dysfunction [102,103].

Clinical presentation varies widely, ranging from nonspecific symptoms to acute heart failure, malignant arrhythmias, or sudden death. Because early symptoms lack specificity, diagnosis generally relies on integration of cardiac biomarkers, electrocardiography, echocardiography, and cardiac magnetic resonance imaging, underscoring the importance of early clinical suspicion and multidisciplinary evaluation [104]. Despite increasing recognition, significant gaps remain in understanding age-specific disease mechanisms and optimal management strategies in pediatric myocarditis, highlighting the need for prospective multicenter studies.

### 5.2 Evolving Diagnostic Strategies

Advances in cardiovascular imaging and biomarker development have progressively shifted the diagnostic approach to myocarditis from reliance on invasive procedures toward integrated noninvasive assessment, a transition particularly evident in pediatric populations. Although cardiac enzymes, electrocardiography, and echocardiography remain essential initial screening tools, their limited specificity has positioned CMR as a central modality in the evaluation of suspected myocarditis. By enabling direct tissue characterization of myocardial edema, necrosis, and fibrosis, and through the application of the updated Lake Louise Criteria incorporating T1 and T2 mapping, CMR provides improved diagnostic accuracy and allows quantitative assessment of inflammatory activity [56,105].

High-sensitivity cardiac troponin testing further contributes to early evaluation; values below defined thresholds demonstrate high negative predictive value and may reduce unnecessary imaging studies [35]. Emerging biomarkers, including circulating microRNAs and inflammatory mediators, are also being investigated as adjunctive tools for early detection and risk stratification [106].

Although EMB remains the histological reference standard, its invasive nature and sampling variability limit routine clinical use. Advances in immunohistochemical and molecular analyses have improved diagnostic yield, particularly in rapidly progressive disease or cases in which tissue confirmation is required to guide immunosuppressive therapy [107]. Consequently, contemporary diagnostic frameworks increasingly emphasize CMR- and biomarker-guided evaluation, reserving EMB for selected high-risk or diagnostically uncertain cases.

## 6. Prognosis and Future Perspectives

### 6.1 Prognostic Determinants

The clinical course of myocarditis is influenced by multiple interacting factors, including etiology, disease severity at presentation, timing of therapeutic intervention, and individual patient characteristics. Outcomes differ substantially across etiologic subtypes. ICI–associated myocarditis carries a particularly high early mortality risk, and markedly elevated cardiac troponin levels together with reduced LVEF are associated with adverse outcomes [108]. Although viral myocarditis frequently presents as an acute illness, long-term follow-up studies indicate a persistent risk of heart failure and major cardiovascular events, especially in patients with histological abnormalities [109].

Clinical presentation also contributes to prognostic assessment. Patients presenting primarily with chest pain generally experience more favorable outcomes, whereas dyspnea, malignant arrhythmias, or circulatory collapse indicate a high-risk disease course. In fulminant myocarditis, elevated lactate levels and renal dysfunction are associated with markedly increased mortality [110,111]. Early therapeutic intervention is therefore critical: prompt initiation of mechanical circulatory support, including ECMO, has been associated with short-term survival in severe cases [112,113], while immunosuppressive therapy or intravenous immunoglobulin may facilitate recovery in selected patients with virus-negative inflammatory myocarditis, although evidence remains largely observational.

Patient-specific factors further modify long-term prognosis. Myocarditis occurs more frequently in men and at younger ages, and genetic susceptibility—such as a family history of dilated cardiomyopathy or heart failure—is associated with poorer long-term outcomes [114,115]. In addition, biomarker and imaging parameters contribute to risk stratification. Elevated NT-proBNP and cardiac troponin levels, together with cardiac magnetic resonance features such as the extent of LGE and impaired left atrial strain, have been associated with increased risks of mortality and cardiovascular events [116,117,118]. Integrated assessment combining etiological classification, clinical presentation, imaging findings, and biomarker profiles therefore provides a framework for individualized risk stratification and management.

### 6.2 Prognostic Assessment Tools

Prognostic evaluation in myocarditis increasingly relies on multimodal integration of imaging, functional assessment, and circulating biomarkers. CMR plays a central role, with LGE representing one of the most consistently reported markers of adverse outcomes; both its presence and extent have been associated with major adverse cardiac events, including death, heart failure, and heart transplantation [119]. Quantitative indices such as native T1, T2 mapping, and ECV provide sensitive detection of myocardial inflammation and fibrosis, and elevated T1 and ECV values have been associated with adverse outcomes in observational studies [120,121].

Myocardial strain imaging further refines functional risk assessment. Reductions in left ventricular GLS and global circumferential strain (GCS), measured by CMR feature-tracking or speckle-tracking echocardiography, have been associated with clinical outcomes, particularly in patients with preserved ejection fraction. Left atrial and right ventricular strain parameters provide additional prognostic information [55,122].

Elevated cardiac troponin and BNP/NT-proBNP levels are consistently associated with worse clinical outcomes, reflecting myocardial injury and hemodynamic stress. Inflammatory markers may provide additional information on disease activity, although their prognostic value remains less well established. In critically ill patients, dynamic indicators such as the vasoactive-inotropic score (VIS) may assist in monitoring circulatory support requirements and recovery trajectories [123,124].

Integration of imaging features, functional parameters, and biomarker profiles enables improved identification of patients at risk for adverse outcomes, supporting individualized follow-up strategies [125,126,127,128].

### 6.3 Future Research Directions

Despite substantial advances, myocarditis remains characterized by complex pathophysiology and marked clinical heterogeneity, leaving important gaps in mechanistic understanding and clinical management. A key priority is to better define the interplay among viral infection, immune dysregulation, and genetic susceptibility. Increasing evidence suggests that genetic variants, particularly in desmosomal genes such as *DSP (desmoplakin)*, may influence inflammatory cardiomyopathy phenotypes and arrhythmic risk, highlighting the need for integrated genomic and immunologic approaches [129,130].

Improving diagnostic precision and individualized therapeutic strategies represents another major objective. Although CMR has become central to noninvasive tissue characterization, further advances are required to enhance disease specificity and prognostic accuracy. Artificial intelligence–driven integration of multimodal datasets, including imaging, genomic, and clinical variables, may enable the development of more robust diagnostic and risk-prediction models and support etiology-directed therapeutic decision-making [131].

Optimization of targeted immunomodulatory therapies also remains an important research direction. While broad immunosuppression continues to form the basis of treatment, pathway-specific interventions and emerging cellular immunotherapies may provide more effective options for refractory disease as mechanistic understanding evolves [24,26]. In addition, the mechanisms and long-term outcomes of vaccine-associated myocarditis require continued investigation, particularly regarding immune mechanisms, genetic susceptibility, and potential structural myocardial sequelae. Multidisciplinary integration of genomics, immunology, advanced imaging, and computational approaches will be essential to translate mechanistic insights into precision clinical care.

## 7. Conclusion

Myocarditis is a clinically heterogeneous inflammatory myocardial disorder in which early recognition and precision management remain challenging. Advances in multimodal imaging—particularly CMR—together with progress in molecular biomarkers and pathogen detection have substantially improved diagnostic accuracy and enabled etiology-oriented risk stratification. Although endomyocardial biopsy remains the histologic reference standard, contemporary diagnostic strategies increasingly emphasize integrated noninvasive assessment.

Supportive therapy continues to form the foundation of management, while etiology-directed immunomodulatory strategies have improved outcomes in selected immune-mediated forms of myocarditis. The expanding availability of mechanical circulatory support has further enhanced survival in fulminant disease, although optimal treatment algorithms and long-term therapeutic effects remain to be fully defined. Individualized diagnostic and therapeutic pathways are especially important in pediatric and other specialized patient populations.

Future progress will depend on the integration of genomic, immunologic, imaging, and computational approaches to refine etiologic classification, improve precision diagnostics, and support individualized treatment strategies. Large multicenter prospective studies and standardized longitudinal follow-up frameworks will be essential to optimize therapeutic decision-making and improve long-term outcomes.
